# You Are Old, but Are You Out? Intergenerational Contact Impacts on Out-Group Perspective-Taking and on the Roles of Stereotyping and Intergroup Anxiety

**DOI:** 10.3389/fpsyg.2022.781072

**Published:** 2022-03-17

**Authors:** Yanxi Long, Xinxin Jiang, Yuqing Wang, Xiaoyu Zhou, Xuqun You

**Affiliations:** ^1^School of Psychology, Shaanxi Normal University, Xi’an, China; ^2^Shaanxi Provincial Key Research Center of Child Mental and Behavioral Health, Xi’an, China; ^3^Shaanxi Provincial Key Laboratory for Behavioral and Cognitive Psychology, Shaanxi Normal University, Xi’an, China; ^4^The Mental Health Education Center, Xuzhou Medical University, Xuzhou, China

**Keywords:** intergenerational relationship, prior contact, imagined contact, perspective-taking, stereotype, intergroup anxiety

## Abstract

Perspective-taking (PT) is an important ability to imagine the world from another’s point of view. Prior studies have shown that younger adults are more likely to consider the opinions of age-based in-group members relative to out-group members. However, the cause of this priority is still unknown. We conducted three independent studies to explore the effect of intergenerational contact on younger adults’ PT toward older adults and the possible roles of stereotyping and intergroup anxiety. A total of 192 college students completed the Perspective taking Scale in Study 1 after being primed with age-based intergroup relationships. The results indicated that younger adults found it more difficult to take the perspective of older adults than that of their peers. 200 college students completed the Prior Contact Scale, Intergroup Anxiety Scale, Negative Stereotype Scale, and PT Scale in Study 2. The results demonstrated that intergenerational contact improved PT toward older adults by disrupting negative stereotypes, and intergroup anxiety moderated this mediating relationship. A total of 215 college students completed the PT Scale in the context of imagining intergenerational contact in Study 3. Interestingly, imagined contact effectively increased younger adults’ ability to take older adults’ perspectives. The present research verifies that contact is important for influencing younger adults’ emotional (intergroup anxiety) and attitudinal (stereotyping) factors that are critical to improving younger adults’ ability to take older people’s perspectives. This is of great significance for developing harmonious intergenerational relationships.

## Introduction

In a family, younger children or younger adults often live together with older adults, especially in an extended family. A younger child can develop their social abilities to enable them to take the perspective of older adults through their contact experience in harmonious family life. Nonetheless, they still seem to hold negative stereotypes toward older adults due to physical differences and psychological distance, and these hinder the communication between these two age-based groups. For example, because of the fact that the older generation is more vulnerable to COVID-19, some younger adults hold negative stereotypes toward the older generation regarding the global pandemic (Madrigal et al., 2021). They even use the phrase Boomer Remover to represent the fact that older adults have higher COVID-19 mortality than younger adults (Meisner, 2020). With regard to social welfare, individuals’ well-being, and cooperation between different generations in the long term, one must pay attention to the development of a harmonious and respectful intergenerational relationship. It is of great significance, therefore, for younger adults to be able to interpret environmental clues regarding stereotypes and take the perspective of older adults if they are to communicate effectively with each other.

Perspective-taking (PT) is the process of “imagining oneself in another’s shoes.” It is a strategy that can reduce stereotyping and prejudice and promote social harmony (Galinsky et al., 2005). It is thought that PT is a powerful tool to improve intergroup relationships (e.g., Galinsky and Moskowitz, 2000; Trötschel et al., 2011), including intergenerational relationships (Keeling, 2012). Less intergenerational contact experience (e.g., Kwong See and Nicoladis, 2010; Lytle et al., 2020), negative stereotypes of older adults (Hargis and Castel, 2019), and a higher level of intergenerational anxiety (Todd et al., 2015) toward older adults may be the reasons that younger adults do not easily take the older generation’s perspective. This may be harmful to intergenerational relationships and aggravate intergenerational conflicts. With regard to contact, researchers believe that contact between groups allows individuals (such as younger adults) to take the perspective of out-group members (such as older adults), which then reduces prejudice (Pettigrew and Tropp, 2008). Conversely, younger adults may have a worse understanding of older adults due to social or geographic distance in daily life (Monserud, 2011). Negative stereotypes toward older adults are that they physically fragile and disabled, have obvious problems with memory deficits (Shepherd and Brochu, 2021) and conservative thoughts, also are poor at multitasking and technology (Weeks et al., 2017). Some younger adults even believe that they occupy jobs, opportunities, and resources that “belong” to the younger generation (Meisner, 2020). These kinds of negative stereotypes may engender younger adults’ anxiety toward the older generation (Fasbender and Wang, 2017), adding to intergenerational prejudice and discrimination.

Prior studies have shown that younger adults’ PT is influenced by life experience (contact), emotional factors (intergroup anxiety), and attitudinal factors (stereotyping). However, the relationships between these factors and the mechanisms beyond them are still unknown. Therefore, the present research aims at understanding how younger adults’ intergenerational contact experiences influence their ability to “stand in older adults’ shoes” (PT), which is critical to intergenerational communication and cooperation. We also investigate the mediating effect of negative stereotypes toward older adults and the moderating effect of intergroup anxiety in the relationship between prior intergenerational contact and PT.

### The Younger Generation Takes the Perspective of Younger Adults More Easily Than That of Older Adults

Prior intergroup relationship research has found that individuals more easily take the perspective of in-groups rather than out-groups (Galinsky and Moskowitz, 2000). This effect is known as in-group favoritism and out-group bias. PT can be used as an efficient tool to increase out-group cooperation (see Bilewicz, 2009; Trötschel et al., 2011). With regard to age-based intergroup relationships, one may argue that the age-based group identity of younger adults may change as they age (Short et al., 2019), indicating that the younger generation and the older generation may become more unified. For example, in the context of Chinese traditional prefigurative culture, the younger generation was a total replication of the older generation in cultural inheritance, and the moral authority spontaneously transferred from the parents to the child when the child became a parent in their own right (Zheng and Chen, 2012). However, the intergenerational relationship research in the modern age had emphasized the independence between generations, making age a standard to separate younger adults and older adults in research (e.g., Meshel and MCGlynn, 2004; Tam et al., 2006; see also Zuo et al., 2007; Chi, 2013; Teater, 2018). Additionally, according to the present situation of intergeneration relationships that we discussed above, it is also necessary for us to consider whether age may separate these two generations due to apparent cognitive and physical differences between them and to the struggle of survival in the context of the COVID-19. Moreover, it had been reasonably supposed by Galinsky et al. (2008) that individuals hold more strongly to stereotypes after taking the perspectives of the targets, no matter who the targets were. They found that individuals utilized stereotypes about the targets in order to form their perspectives (Galinsky et al., 2005), which in turn increased the self-other overlap through the process of including the other’s stereotypical traits in the self (Galinsky et al., 2008). These empirical studies give us grounds to put forward our assumption that younger adults will try to utilize their negative stereotypes about older adults in order to take their perspective, but only to find it difficult to proceed because of the negative traits (e.g., conservative thoughts) they imposed on older adults. On the other hand, because of the similarity of their peers, they find it easier to take the perspectives of younger adults. For example, young children associate with peers by using age information (Yoo, 2015).

All in all, the younger generation will act differently toward their peers (the age-based in-group) than to older adults (the age-based out-group) in their PT. Specifically, the current research expects a pattern of PT differential in intergroup relationships; in that, younger adults may find it more difficult to take the perspective of older people than that of younger people. Based on the aforementioned literature reviews and assumptions, the present research expects there to be an in-group favoritism effect in intergenerational relationships; thus, we propose our first hypothesis to be tested in Study 1:

*H1: The younger generation will take the perspective of younger adults more easily than that of older adults.*

### Negative Stereotypes Disrupt the Younger Generation’s PT Toward Older Adults

To our present knowledge, many prior studies have focused on the impact of PT on stereotyping (see Aberson and Haag, 2007; Wang et al., 2018; Baryshevtsev et al., 2020), but few have considered the reverse (see Lin et al., 2021). In the field of intergenerational research, only one published study has analyzed the influence of stereotypes about older adults on PT. Hargis and Castel (2019) asked younger participants to estimate the memory task accuracy of older participants before starting the same task themselves. It was found that the younger participants overestimated the performance of the older participants. The researchers assumed that this was because young adults lacked memory information about the older participants and had to refer to their stereotypes toward old people to estimate, which in turn impaired their PT toward old participants. Although there is an urgent need for relevant empirical evidence in the field of intergenerational relationships, current intergroup relation research suggests that individuals may take the out-groups’ perspective under the influence of out-group stereotypes (Galinsky et al., 2008). The same mechanism may also occur in the process of the younger generation taking the perspective of the older generation, meaning that younger adults will imitate the targets’ state of mind according to their negative perceptions toward older adults (e.g., lower memory capacity and stubbornness) and will react correspondingly on this basis. In this point of view, younger adults will have difficulties in taking older adults’ perspectives due to the conflict between actual and perceived older adults’ abilities. The second hypothesis of the current research is thus proposed:

*H2: The younger generation’s negative stereotypes toward older adults will reduce their ability to take the older generation’s perspective.*

### Impacts of Intergenerational Contact on PT and the Mediating Role of Stereotyping

Prior research has reached a consensus that intergroup contact effectively promotes PT toward out-group members (see Aberson and Haag, 2007; Swart et al., 2011; Teater and Chonody, 2015; Mana, 2019). Pettigrew and Tropp (2008) used a meta-analysis to verify that intergroup contact increases individuals’ PT toward out-group members. This conclusion has also been found to be valid for research in the field of intergenerational relationships. For example, Fair and Delaplane (2015) conducted a longitudinal study, namely the Elementary Students’ Intergenerational Service Learning Project, in which second-graders were asked to visit older adults monthly and connect with them in retirement facilities. This research found that children became more empathetic and became more readily able to take the older adults’ perspective at the end of the project. Unfortunately, there is little additional empirical evidence to verify the influence of contact toward PT in intergenerational relationships. We thus propose the third hypothesis:

*H3: The more prior intergenerational contact younger adults have experienced, the more easily they will take the older generation’s perspective.*

Many social psychologists agree with the view that the more individuals contact out-group members, the fewer stereotypes will be activated during contact (e.g., Galinsky and Moskowitz, 2000). Earlier studies revealed that younger adults who frequently contacted older people had more precise information and a more positive attitude toward them, showing an out-group favoritism effect (Jiang and Zhou, 2012; Hock and Mickus, 2019; Cesnales et al., 2020). Madrigal et al. (2021) used a convergent parallel mixed method design to study the impact of an art installation on intergenerational relationships, and they found that young adults’ negative stereotypes about older adults turned into positive perceptions. Keeling (2012), working with young rural students from New Zealand, suggested that teenagers who have more face-to-face contact experience with their grandparents would evaluate their grandparents more positively and maintain a more intimate relationship with them. On the other hand, if the younger generation kept avoiding interactions with the older generation, then the younger would misinterpret the older in many ways (Weeks et al., 2017), which in turn would intensify negative stereotypes. Blieszner and Artale (2001) conducted mixed-method research to investigate how intergenerational contact changes the younger adults’ misperceptions about older adults. More than half of the participants demonstrated that interactions and connections between themselves and older people bridged the generational gap and increased their understanding of older people. It can be seen from the research results above that prior contact can effectively relieve and eliminate younger adults’ negative stereotypes and prejudice toward older adults.

Integrating the hypotheses that negative stereotyping disrupts PT (H2), the view that intergenerational contact may improve younger adults’ PT toward older adults (H3), and the results that contact decreases the level of negative stereotypes, the present research thus investigates the mediating role of negative stereotypes in the relationship between prior contact and PT (H4).

*H4: The younger generation’s prior contact with older adults will have an impact on out-group perspective-taking through the mediation of negative stereotypes.*

### The Moderating Role of Intergroup Anxiety

Intergroup anxiety reflects the extent of a person’s negative experiences, such as nervousness, awkwardness, and even hostility, when engaging with out-groups or anticipating such engagement (Stephan, 2014). Several studies have indicated that intergroup anxiety decreases the level of individuals’ engagement intention toward out-groups and thus triggers negative reactions in intergroup encounters. This is an obstacle to effectively interacting with out-groups and damages intergroup trust (Plant and Devine, 2003; Aberson and Haag, 2007).

As an affective variable, intergroup anxiety has been considered to influence the relationship between prior contact and PT. Anxiety decreases the ability to take others’ perspective and increases the tendency toward egocentricity (Todd et al., 2015). In other words, if a person encounters an out-group member and feels more anxious than they would with an in-group member, then this negative emotion may impede their ability to take the perspective of the out-group member.

As for the relationship between prior contact and stereotyping, a number of prior studies have focused on the mediating role of intergroup anxiety rather than moderation (see Abrams et al., 2006; Hutchison et al., 2010; Fasbender and Wang, 2017). These results are consistent with the Intergroup Anxiety Theory (Stephan, 2014), which proposes that intergroup anxiety is a mediator between its antecedents (i.e., prior contact) and consequences (i.e., out-group stereotyping). Stephan (2014) also mentioned that this mediated causal relationship is reciprocal, which means intergroup anxiety may also have an impact on its antecedents, and its consequences may also have an impact on it. In addition to the view that a higher level of intergroup anxiety is related to lower levels of contact and negative stereotypes (no matter what the directions of these complex relations are), one could propose that intergroup anxiety has another potential function to influence the relationship between variables besides mediation, namely, moderation. For example, Reimer et al. (2021) conducted a field study to verify the effectiveness of contact-based intergroup relationship intervention and found that positive contact indeed decreased intergroup anxiety and increased out-group PT but not intergroup attitudes. This result offers a possibility that a lower level of intergroup anxiety may reduce the influence of contact toward out-group attitudes. Few studies have investigated whether different levels of intergroup anxiety have different impacts on the relationship between prior contact and stereotype, especially in the intergenerational domain. Thus, one of the aims of the present study is to investigate the potential moderating role of intergroup anxiety between prior intergenerational contact and stereotypes of older adults.

In all, the present research assumes that intergroup anxiety moderates the relationships between stereotypes and PT. Considering the complex relationships among prior contact, negative stereotypes, intergroup anxiety, and PT that were discussed above, we propose the fifth hypothesis.

*H5: Intergroup anxiety will moderate the relationships between prior contact and perspective-taking (H5a), between prior contact and stereotyping (H5b), and between stereotyping and perspective-taking (H5c).*

**FIGURE 1:**
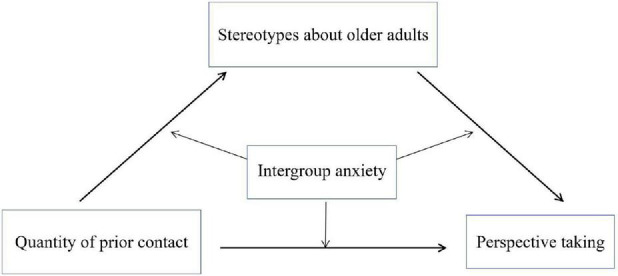
The hypothetical model diagram.

The aim of Study 2 in the current research is to construct a moderating mediation model to test H2 through H5. To be more specific, in the hypothetical model (see Figure 1), prior contact influences PT through the mediation of negative stereotypes, and intergroup anxiety moderates all three pathways in the mediating relationships.

### Impacts of Imagined Intergenerational Contact on Perspective-Taking

According to the discussion above, intergenerational contact may have an impact on PT; we have tested this in Study 1 and Study 2. However, from the perspective of research intervention, it is difficult to change individuals’ experiences toward intergenerational contacts that happened in the past. It is better and more efficient to intervene in this process through the human imagination. Imagined intergroup contact is the mental simulation of social interaction with a member or members of an out-group category (Crisp and Turner, 2009), and it is a wonderful opportunity to seek intergroup connection across time and space. Abundant research has shown that the positive-imagined intergroup contact encourages positive intergroup relationships (e.g., Bilewicz and Kogan, 2014; Ioannou et al., 2015; Vezzali et al., 2015), and the same truth applies to intergenerational relationships (e.g., Prior and Sargent-Cox, 2014). There is rich evidence regarding whether and how younger adults change their attitudes toward older adults after imagining positive age-based out-group interaction. For example, Chen et al. (2016) found that younger adults who imagined positive encounters with older adults will perceive older adults more positively. The research of Harwood et al. (2015) demonstrated that the positive-imagined intergenerational contact not only improved younger individuals’ positive perceptions of older adults but also increased their intention to communicate with older adults in the future. Unfortunately, few empirical studies have investigated whether the younger adults’ PT ability toward older people improves after imagining positive intergenerational contact. Thus, the present study considers the perspective of younger adults toward positive intergenerational encounters, using an imagined contact scenario paradigm (Study 3) to investigate how the younger adults’ PT ability toward older people is changed. We thus posit the following hypothesis:

*H6: The younger generation will take older adults’ perspective more easily after imagining positive contact with the older generation.*

All in all, the present research conducts three independent studies to examine the factors that affect intergenerational relationships. Specifically, Study 1 aims to confirm differences in younger adults’ PT toward different generations, Study 2 constructs a moderating mediation model to investigate the influence of younger adults’ prior contact on age-based out-group PT, and Study 3 examines the intervention effectiveness of imagined contact on PT.

## Study 1: The Differential of PT Toward Age-Based Intergroup Targets

### Methods

#### Participants

A power analysis conducted in G*power (version 3.1.9.7) indicated that the minimum required total sample size was *N* = 210 (with each group having a sample size of 105) to achieve a sufficient power (1 − β = 0.95) with a medium effect size of Cohen’s *d* = 0.50. We then recruited 192 college students (*M*_*age*_ = 21.90, SD = 2.66) from a university in Shaanxi Province as participants for the current study and randomly assigned them to either the age-based in-group (whose interaction target was younger adults, *N* = 92, 25 men) or the age-based out-group (whose interaction target was older adults, *N* = 100, 15 men).

#### Instruments

##### Perspective-Taking

The PT Subscale of Davis’s (1980) Interpersonal Reactivity Index was used to measure participants’ PT. Individual scale items were revised to match the interaction targets. The targets were the younger generation in the age-based in-group and the older generation in the age-based out-group. There were seven items on the scale (e.g., “I try to look at older/younger adults’ side of a disagreement before I make a decision”). Response options were 1 = *not at all* to 5 = *very much*. Higher total scores indicated that individuals more easily took the other group’s perspective. The internal consistency of the scale was 0.75. The Cronbach’s α for the in-group was 0.78, and the Cronbach’s α for the out-group was 0.71.

#### Procedure

**FIGURE 2:**
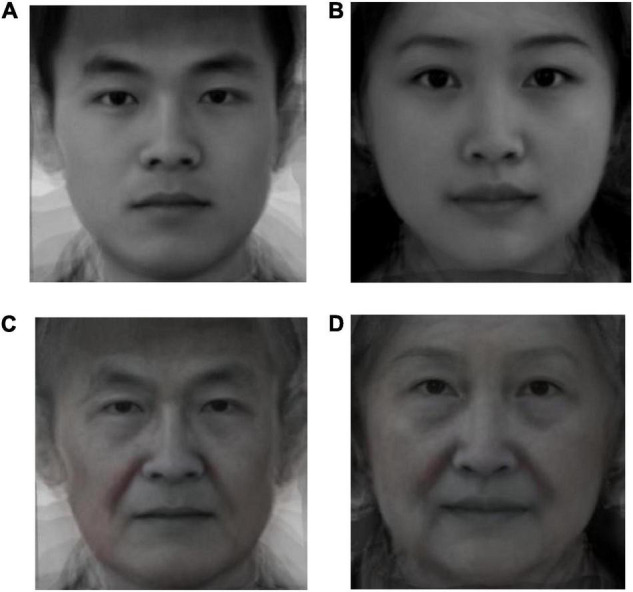
Facial images for different age groups. Pictures **(A,B)** were the priming facial pictures that were shown to participants in the age-based in-group. Pictures **(C,D)** were the priming facial pictures that were shown to participants in the age-based out-group. Images sourced from Hou et al. (2019) (CC-BY).

Before conducting the formal process of filling out questionnaires, all participants in both the age-based in-group and the out-group were asked to view two human facial pictures (a male and a female, see Figure 2) for 30 s to prime age-based intergroup relationships. This was done according to the priming paradigm of Ofan et al. (2011), which holds that simply showing facial images of different races can evoke participants’ evaluative associations with race. Hudson et al. (2019) similarly effectively primed participants’ perception toward race-based intergroup relations through the assessment of racial identity after demonstrating different race facial images. These results indicate that salient visual characteristics of facial images may increase the salience of intergroup relations (e.g., for race). Like race, age is also included in the “Big Three” of cues by which people process social categorization (Zuo et al., 2019). Age information of facial images can also be rapidly extracted (Rhodes and Anastasi, 2012). This demonstrates a robust intergroup effect of the own-age bias (OAB), the phenomenon by younger adults can better recall facial images of an age-based in-group (i.e., younger adults) than of an out-group (i.e., older adults) (Short et al., 2019). In all, it is reasonable to prime younger adults’ perception toward intergenerational relations by demonstrating facial images of different ages.

Thus, in the current study, participants in the in-group relationship condition viewed two younger adults’ facial images (see Figures 2A,B), whereas participants in the out-group relationship condition viewed two older adults’ facial images (see Figures 2C,D). This drew their attention to the age-based group boundaries. Then, they completed the questionnaires. The facial images of younger adults used in the study of Hou et al. (2019) were adopted in the current study. All facial images are composite images composed of four standard human faces from Hou’s research (Hou et al., 2019), indicating that all the facial images used in the current research were not taken from real persons. We then used a professional photo filter software to age the figures to obtain the facial images of older adults that were required. All facial images were unbiased processed. In order to test the validity of this manipulation, we added a question for assessing the similarity of identity in age between participants and the targets in the priming pictures through 1 = *not at all* to 7 = *very much*. Higher scores indicated that the younger adults had a higher level of age identification with the targets.

### Results

#### Validity Test of Priming for Age-Based Intergroup Relationships

An independent-sample *t*-test showed that the identity similarity scores of the age-based in-group participants (*M* = 3.87, SD = 1.49) were significantly higher than those for the out-group participants (*M* = 2.67, SD = 1.80), *t* = 5.01, *p* < 0.001, Cohen’s *d* = 0.73, indicating that facial pictures of different generations effectively aroused age-based intergroup relationships in the present participant cohort.

#### Tests of Differential in Perspective-Taking

Preliminary analysis showed that age and gender were not correlated to PT of either the in-groups or the out-groups. In case age and gender potentially influenced the core variable (PT), both demographic variables were still controlled as covariates. UNIANOVAs showed that the PT scores for the age-based in-group (*M* = 26.50, SD = 4.08) were significantly higher than those for the out-groups (*M* = 24.87, SD = 3.90), *F* = 8.07, *p* = 0.005, η^2^ = 0.04. This result supports H1, indicating that compared to age-based in-groups, younger adults find it more difficult to take older adults’ perspective. This appears to be an example of the phenomenon of the generation gap.

### Discussion

The current study verified the differentiation of younger adult PT toward age-based in-group and out-group targets. Specifically, younger adults took the perspective of the younger generation more easily than that of the older generation, which implies that misunderstanding and misperception may exist in age-based intergroup relationships. The mechanism behind the differential thus needed further investigation.

## Study 2: The Impact of Prior Contact on Age-Based Out-Group PT: The Mediating Role of Negative Stereotypes and the Moderating Role of Intergroup Anxiety

### Methods

#### Participants

Participants were recruited from a university in Shaanxi Province. There were 200 college students (*M*_age_ = 21.54, SD = 3.07, 34 men) who took part in the survey. Because of the imbalanced ratio of gender in this case, we gave special attention to analyzing and controlling the potential influence of gender in follow-up analyses.

#### Instruments

##### Prior Contact

The quantity of contact subscale was extracted from the prior contact scale of Husnu and Crisp (2010) to measure younger adults’ previous contact with older adults. The current study also made an adaption to item statements to match the PT target of older adults. There were four items: “How many older adults do you know?”; “In everyday life, how often do you encounter older adults?”; “In everyday life, how frequently do you interact with older adults?”; and “In everyday life, how much contact do you have with older adults?” All items above used a scale from 1 = *none* to 7 = *a lot*. Higher total scores indicated that individuals had a higher level of contact with older adults. The Cronbach’s α was 0.88 in the current study.

The current study also extracted one particular item, “unpleasant–pleasant,” in the subscale of the quality of contact to categorize different types of prior contact according to the contact valence. This item also used a 7-point Likert scale. Scores higher than four points represented positive contact and lower than four points represented negative contact. Accordingly, the current study could also investigate whether the valence of prior contact influences younger adults’ PT toward older adults, but the potential mediating or moderating functions of quality of contact were not addressed in the present study.

##### Intergroup Anxiety

The Intergroup Anxiety Scale developed by Stephan and Stephan (1985) was used to measure the level of anxiety felt when younger adults contact older adults. There were 10 adjective terms for describing feelings of interaction with out-group members, including seven negatively valenced adjectives (e.g., awkward and irritated) and three positively/reversed-scored adjectives (happy, accepted, and confident). Participants were asked to evaluate their corresponding feelings from 1 = *not at all* to 7 = *very much*, according to their experience of interactions with older adults. Higher total scores demonstrated that individuals had experienced a higher level of anxiety when they interacted with older adults. The Cronbach’s α was 0.70 in the present study.

##### Negative Stereotype

The self-stereotyping scale adapted by Dang et al. (2020) was used to measure younger adults’ negative stereotypes toward the older generation. The first-person appellations (i.e., *I*) in the items were all changed to third-person appellations (i.e., *older adults*) to adjust the scale to the needs of the current study. There were 10 items on the scale (e.g., “Many older adults are just living in the past”). Response options were 1 = *not at all* to 4 = *very much*. The higher total scores illustrated a higher level of negative stereotypes toward older adults. The internal consistency of the scale in the research of Dang et al. (2020) was 0.70, and in this study, it was 0.79.

##### Perspective-Taking

The current study used the same scale in Study 1 to measure PT. The Cronbach’s α was 0.73 in this study.

#### Procedure

Because Study 2 focused on younger adults’ cognition (PT), personal experience (prior contact), emotion (intergroup anxiety), and attitude (stereotypes) toward the older generation, all participants were asked to view two facial pictures of older adults (the same male and female pictures, which were used in Study 1) to activate their perceptions of age-based out-group relations before filling out the questionnaires. Once again, the identity similarity was evaluated by a 7-point Likert scale. The PT scale, the intergroup anxiety scale, the older adults’ stereotype scale, and the prior contact scale were completed in turn.

#### Data Analysis

Study 2 aimed at unveiling the mechanism by which younger adults’ previous contact affects their PT toward older adults by analyzing the pathways in the hypothetical model. To verify the second to the sixth hypotheses suggested in the introduction, Study 2 constructed a moderating mediation model. The mediator in the relationship between prior contact and PT was stereotyping, and the moderator was intergroup anxiety. The present study used SPSS 21.0 to conduct ANOVAs and other statistical methods to analyze the collected data.

### Results

#### Preliminary Analyses

The results of the one-sample *t*-test showed that the scores of similarity were significantly lower than the average point, which was 4, *M* = 3.00, SD = 1.83, *t* = − 7.76, *p* < 0.001, Cohen’s *d* = 0.55. These results indicated that the participants had a low level of age identification toward the older adults in the facial images.

Mean and SD of all measured variables are presented in Table 1A. Due to the fact that gender was significantly related to stereotypes of older adults and intergroup anxiety, an independent-sample *t*-test was conducted. The results indicated that male younger adults had more negative stereotypes (*M*_male_ = 26.56, SD_male_ = 5.95, *M*_female_ = 24.42, SD_female_ = 4.65, *t* = 1.97, *p* = 0.06, Cohen’s *d* = 0.03) and more anxiety (*M*_male_ = 39.85, SD_male_ = 9.91, *M*_female_ = 34.91, SD_female_ = 6.05, *t* = 2.80, *p* < 0.001, Cohen’s *d* = 0.07) toward older adults compared to women younger adults. Thus, in the following statistical analyses, gender was controlled as a covariate. In the case of a potential influence of age toward the core variables, age was controlled as well.

**TABLE 1A:** Descriptive statistics and correlations between variables.

Variables	M	SD	1	1a	1b	2	3	4	5	6
1. Prior Contact	21.26	5.60	1							
1a. Quantity of Contact	16.25	4.93	0.98***	1						
1b. Quality of Contact	5.01	1.18	0.64***	0.48***	1					
2. Stereotypes	24.79	4.95	−0.15*	−0.14*	–0.13	1				
3. Intergroup Anxiety	35.75	7.08	0.002	0.01	–0.05	0.49***	1			
4. Perspective-Taking	25.03	4.08	0.26***	0.22***	0.31***	−0.25***	−0.22**	1		
5. Gender			0.06	0.03	0.13	−0.16*	−0.26***	0.11	1	
6. Age	21.54	3.07	–0.10	–0.09	–0.13	0.12	0.01	–0.03	−0.03	1

*p < 0.05, **p < 0.01, ***p < 0.001.

The correlation results revealed that stereotypes of older adults were negatively related to PT (*r* = − 0.25, *p* < 0.001), which suggests that younger adults who held more negative stereotypes had more difficulties in taking older adults’ perspective. As expected, the quantity of contact was positively related to PT (*r* = 0.26, *p* < 0.001) and indicated that younger adults who had more prior contact with older people found it easier to take the older generation’s perspective. Moreover, the quantity of prior contact was negatively related to stereotypes of older adults (*r* = − 0.15, *p* = 0.03), which indicated that younger adults who had less prior contact held more negative stereotypes toward older people.

#### The Mediating Role of Stereotyping

The current study conducted stepwise regression and bootstrap analysis to test the mediation effect of negative stereotypes of older adults while controlling gender and age (see Table 1B). The results of equation 1 show that younger adults who previously had more contact with older adults were more easily able to take older adults’ perspectives; this supports H3. The results of equation 2 also showed that younger adults who had more contact experience with older adults held a lower level of negative stereotypes. The results of equation 3 not only verified that younger adults who had more negative stereotypes toward older adults found it more difficult to take their perspective (supporting H2) but also confirmed the mediating role of stereotypes in the relationship between prior contact and PT (supporting H4). Specifically, younger adults who previously had had less contact experience with the older generation held more negative stereotypes toward them, which consequently disrupted the ability of younger adults to take the perspective of older people.

**TABLE 1B:** The mediation effect of stereotypes.

	Equation 1 (Perspective-taking)				Equation 2 (Stereotypes)				Equation 3 (Perspective-taking)			
	β	*t*	95%CI		β	*t*	95%CI		β	*t*	95%CI	
			*LL*	*UL*			*LL*	*UL*			*LL*	*UL*
**Variables**												
Quantity of contact	0.22	3.16**	0.05	0.32	–0.13	–1.83	–0.31	0.04	0.19	2.82**	0.03	0.28
Stereotypes									–0.23	−3.29**	–0.30	–0.07
Gender	0.10	1.50	–0.20	2.45	–0.41	−2.23*	–4.17	0.17	0.19	1.03	–0.62	2.12
Age	–0.01	–0.11	–0.30	0.19	0.03	1.42	–0.01	0.51	0.04	0.19	–0.23	0.20
**Statistics**												
*R* ^2^	0.06				0.05				0.10			
*F*	4.25*				3.77*				5.70***			

*p < 0.05, **p < 0.01, ***p < 0.001.

#### The Moderated Mediating Role of Intergroup Anxiety

**FIGURE 3:**
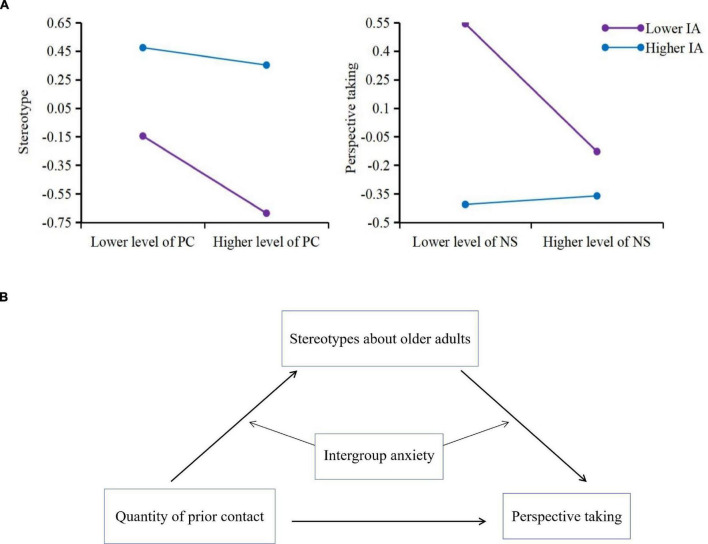
**(A)** Simple slope diagrams in the practical model. PC, prior contact; NS, negative stereotyping; IA, intergroup anxiety. The diagram on the left presents the moderating effect of intergroup anxiety toward the relationship between prior contact and negative stereotypes of older adults, whereas the diagram on the right presents the moderating effect of intergroup anxiety toward the relationship between negative stereotypes of older adults and PT. **(B)** The practical model diagram.

We used the SPSS-PROCESS v3.3 plug-in unit (Model 59) to analyze the moderating role of intergroup anxiety in the hypothetical mediation model while controlling gender and age (see Table 1C). The results of equation 1 demonstrated that the interaction of prior contact and intergroup anxiety significantly predicted negative stereotypes of older adults (β = 0.10, *t* = 2.29, *p* = 0.02). We then conducted a simple slope analysis by dividing intergroup anxiety into higher (one SD above the mean) and lower (one SD below the mean) level groups to further test the moderated mediation effect (see Figure 3). The results showed that the mediation effect of a lower level of intergroup anxiety was − 0.27, 95% CI = (−0.44, − 0.11), which implied that a lower level of intergroup anxiety moderated the negative prediction of prior contact on stereotyping, meaning that, in the condition of low intergroup anxiety, younger adults who previously had less contact with older adults held more negative stereotypes of older adults compared to those who had more frequent contact. Meanwhile, in the condition of high intergroup anxiety, younger adults held more negative stereotypes regardless of whether they had more or less contact with older adults.

**TABLE 1C:** The moderated mediation effect of intergroup anxiety.

	Equation 1 (Stereotypes)			Equation 2 (Perspective-taking)		
	β	*t*	*p*	β	*t*	*p*
**Effects**						
Quantity of contact	–0.17	−2.69**	0.01	0.12	1.70	0.09
Intergroup anxiety	0.41	5.90***	<0.001	–0.30	−3.34***	0.001
Quantity of contact × Intergroup anxiety	0.10	2.29*	0.02	–0.05	–0.82	0.41
Stereotypes				–0.16	−2.05*	0.04
Stereotypes × Intergroup anxiety				0.18	3.79***	<0.001
Gender	–0.06	–0.38	0.70	0.17	0.92	0.36
Age	0.03	1.69	0.09	0.01	0.45	0.65
**Statistics**						
*R* ^2^	0.29			0.18		
*F*	16.22***			6.98***		

*p < 0.05, **p < 0.01, ***p < 0.001.

The results of equation 2 showed that intergroup anxiety negatively predicted PT (β = −0.30, *t* = −3.34, *p* = 0.001), which indicates that younger adults who had a higher level of intergroup anxiety had more difficulty taking older adults’ perspective than those who had a lower level of anxiety. In addition, the interaction of stereotypes of older adults and intergroup anxiety positively predicted PT (β = 0.18, *t* = 3.79, *p* < 0.001). Simple slope analysis (using the same method that was mentioned above to divide the participants into groups with a higher versus lower level of intergroup anxiety) illustrated that the mediating effect of low intergroup anxiety was − 0.34, 95% CI = (−0.52, −0.16). Together, we inferred that, in the condition of low intergroup anxiety, younger adults who held a lower level of negative stereotypes took older adults’ perspective more easily. However, in the condition of high intergroup anxiety, the results showed a similar pattern to the impact of prior contact on stereotyping, meaning that younger adults who held a higher or a lower level of negative stereotyping would always find it difficult to take older adults’ perspective.

The moderating effect of intergroup anxiety on the relationship between prior contact and PT failed to meet the level of significance. In sum, the results for the moderated mediation effect partially supported H5. In particular, intergroup anxiety moderated the relationships between prior contact and stereotyping (H5b) and between stereotyping and PT (H5c), but not between prior contact and PT (H5a). The practical moderated mediation model is presented in Figure 3B.

#### Impact of Quality of Prior Contact on PT

According to the valence of prior contact, we divided participants into a positive contact group (*N*_positive_ = 145), a negative contact group (*N*_negative_ = 21), and a neutral contact group (*N*_neutral_ = 34). UNIANOVA results showed that the main effect of valence was significant (*F* = 4.31, *p* = 0.02, *?*^2^ = 0.04). *Post hoc* tests indicated that the PT of the positive contact group (M_positive_ = 25.52, SD = 4.14) was significantly higher than that of the negative contact group (*M*_negative_ = 23.19, SD = 4.00, *p* = 0.01, Cohen’s *d* = 0.57) and marginally higher than that of the neutral contact group (*M*_neutral_ = 24.06, SD = 3.37, *p* = 0.06, Cohen’s *d* = 0.39). There were no statistically significant differences between the negative contact group and the neutral contact group (*p* = 0.44). These results demonstrated that positive prior contact improved the ability of younger adults to take older adults’ perspective.

### Discussion

In the current study, we constructed a moderating mediation model to examine the impact of prior contact on PT through different functions of stereotyping and intergroup anxiety. We found it important to note that younger adults may take older adults’ perspectives based on their negative stereotypes toward them. Specifically, younger adults’ negative stereotypes toward older adults decreased their ability to take the older generation’s perspective. These empirical findings indicate the importance of younger adults’ prior contact with the older generation. This is consistent with the view of Verhage et al. (2021) that younger adults’ prior contacts may show a unique contribution to understand the outcomes of intergenerational contact.

We then investigated the mediating role of stereotyping. The more prior intergenerational contact younger adults experienced, the lower the level of negative stereotypes they held toward older adults. Furthermore, we found that negative stereotypes mediated the relationship between prior contact and PT. The moderating role of intergroup anxiety was also tested; the results partially supported H5; in that, intergroup anxiety influenced the relationships between prior contact and stereotyping (H5b) and between stereotyping and PT (H5c), but not between prior contact and PT (H5a).

Combining the results of correlation analyses, regression analyses, and variance analyses, we could also infer that a higher quantity of contact and of positive contact positively predicted a higher level of PT. This indicated that a higher quantity of contact and a more positive quality of contact improved the ability of younger adults to take older adults’ perspective. These results were consistent with those of Aberson and Haag (2007). The present study not only showed that intergroup contact will not necessarily facilitate PT unless it has a positive valence and high frequency but also expanded this point of view to intergenerational relationships. It is insightful to learn that the properties of prior contact may be critical to developing harmonious intergenerational relationships through PT, which is a unique process in human–social interaction.

In all, the results of Study 2 demonstrated that a high quantity of positive intergenerational contact improved the ability of younger adults to take older adults’ perspective. However, we cannot change past experience; in that, the quantity and quality of prior intergenerational contact cannot be changed. The manipulation of positive imaginary intergenerational contact seems more practical.

## Study 3: The Impact of Imagined Intergenerational Contact on Age-Based Out-Group PT

### Methods

#### Participants

A power analysis conducted in G*power (version 3.1.9.7) indicated that a minimum sample size of *N* = 220 was required to achieve a sufficient power (1 − β = 0.95) with a medium effect size of *f* = 0.20. Participants were recruited from two universities in Shaanxi Province. A total of 215 college students (*M*_age_ = 21.06, SD = 1.75, 131 men) took part in the survey and were randomly assigned to either the imagined contact group (*N*_imagined_ = 107) or the control group (*N*_control_ = 108).

#### Manipulation of Imagined Intergenerational Contact

The current study employed an imagined depiction task, asking the participants to depict their imagined pictures of positive interactions with an older adult (imagined intergenerational contact group) or beautiful scenery (control group) for 3 min. After imagining, participants in the imagined contact group were asked to write down as many words as possible that portray older adults’ positive traits, whereas the control group was asked to write down as many words as possible that portray the sight of beautiful scenery. The researchers recruited three well-trained psychology major undergraduate students to assess the content’s consistency of both groups’ answers. The inter-rater reliability was acceptable (Cronbach’s α = 0.78), which indicated that both groups indeed followed the requirements to imagine corresponding events and wrote down corresponding words. In other words, the manipulation of imagined intergenerational contact was effective and reliable.

#### Instruments

##### Perspective-Taking

The same PT scale that was used in Study 1 and Study 2 was also employed in the current study, although with some adaptation to adjust for the manipulation of imagined intergenerational contact. The terms that describe the frequency of events (e.g., *usually*, *sometimes*, and *often*) in the original scale were not suitable for the current study. Therefore, the authors deleted all this type of term and added the word *now* in each statement to properly emphasize the imagined contact events’ impact on PT (e.g., “I try to understand older adults better now by imagining how things look from their perspective”). The adapted scale had an acceptable internal consistency (Cronbach’s α = 0.64), and confirmative factor analysis demonstrated a good validity (χ^2^ = 27.98, χ^2^/*df* = 2.00, RMSEA = 0.07, CFI = 0.92, TLI = 0.88, and SRMR = 0.05).

##### Stereotypes and Intergroup Anxiety

We also measured stereotypes of older adults (Cronbach’s α = 0.80) and intergroup anxiety (Cronbach’s α = 0.72) by using the same scales that were employed in Study 2. In the statistical analyses conducted below, we controlled stereotypes of older adults and intergroup anxiety as covariates.

### Results

#### Preliminary Analysis

Descriptive statistics, correlations, and independent-sample *t*-test results are shown in Table 2. None of the three variables had statistically significant differences between the two participant groups. However, the results of correlation analysis demonstrated that both stereotyping and intergroup anxiety correlated with PT in similar ways (see Table 2). In the imagined group, both stereotyping and intergroup anxiety were significantly correlated to PT in ways similar to those that were presented at Study 2. In the control group, stereotyping was still positively correlated with PT, and intergroup anxiety was still negatively correlated with PT. However, the correlation between stereotyping and PT failed to reach the level of significance. Because the aim of Study 3 was to investigate the intervention effect of positive-imagined intergenerational contact toward PT, it was not our intention to include the variables (which in this case were stereotyping and intergroup anxiety) that may influence the mechanism of this process. Therefore, we controlled stereotype and intergroup anxiety as covariates.

**TABLE 2:** The results of descriptive statistics, correlations, and differential analysis.

	Imagined group				Control group					
Variables	M ± SD	Correlations			M ± SD	Correlations			*t*	Cohen’s *d*
		1	2	3		1	2	3		
1. Stereotypes	24.38 ± 3.47	1			24.07 ± 4.41	1			0.57	0.08
2. Intergroup anxiety	28.91 ± 6.83	0.40***	1		28.84 ± 7.66	0.22*	1		0.07	0.01
3. Perspective-taking	25.58 ± 2.99	−0.40***	−0.33***	1	25.24 ± 3.67	–0.12	−0.32***	1	0.75	0.10

*p < 0.05, ***p < 0.001.

#### Test of Differential in Perspective-Taking

After controlling stereotypes of older adults and intergroup anxiety, we conducted UNIANOVA to test the differentiation between the PT scores of the two groups. The results showed that the PT of the imagined contact group (*M*_imagined_ = 26.05, SD = 3.53) was significantly higher than that of the control group (*M*_control_ = 24.60, SD = 3.88), *F*(1, 73) = 5.24, *p* = 0.03, η^2^ = 0.05. This indicated that, compared with the participants who imagined scenery, younger adults who imagined positive intergenerational contact took the perspective of older adults more easily, supporting H6.

### Discussion

The present study aimed at examining the impact of imagined contact on PT toward age-based out-group targets. More specifically, the intergenerational relationship was significantly improved through increased PT after younger adults imagined positive encounters. This result was consistent with prior research (e.g., Harwood et al., 2015; Chen et al., 2016). Imagined intergroup contact is considered to be an effective way to improve out-group PT (see Husnu and Crisp, 2015). It is similarly effective for intergenerational relationships. As argued above, human imagination makes a great contribution to the shaping process of individuals’ perceptual references to other social groups (Crisp and Turner, 2009). The imagination can even make individuals believe that imagined positive encounters with out-groups truly happened in real life (Frye and Lord, 2009). Interestingly, the correlations between stereotypes of older adults, intergroup anxiety, and PT in the current study showed similar patterns to those demonstrated in Study 2, which also indirectly proved the idea that imagined contact has an impact on intergroup relationships in the same way as authentic contact. In this case, younger adults may benefit from imagined contact with older adults that improves their cognition (PT), and thus improves intergenerational relationships.

## General Discussion

The present research conducted three independent studies to investigate the impact of intergenerational contact on younger adults’ PT toward older adults. The goal of Study 1 was to examine whether age-based intergroup relations influenced younger adults’ PT toward the older generation. Study 2 aimed at investigating the mechanism by which prior contact affects PT toward older adults through the mediating role of negative stereotypes of older adults and the moderating role of intergroup anxiety. Study 3 was an attempt to intervene in younger adults’ perceptions of intergenerational relationships by imagining positive contact with older adults. All hypotheses were supported or partially supported, indicating that intergenerational contact is an important tool for influencing younger adults’ cognitive (PT), emotional (intergroup anxiety), and attitudinal (stereotyping) factors that are critical to developing harmonious intergenerational relationships.

The results of Study 1 verified a social situation that cannot be ignored. That was to say, the younger generation had more difficulties in taking the older generation’s perspective, confirming the existence of the phenomenon called the *generation gap*. Although in Chinese traditional culture, the Confucian principles of filial piety shape Chinese younger adults’ more positive attitudes toward older adults to a greater extent than is the case for younger adults in Western culture (Tan and Barber, 2020), the generation gap still exists in daily life. This gap probably comes from the nature of social classification. Based on the social identity theory, individuals prefer to integrate their self-concept into the social group they identify with, classifying themselves as part of the in-group (Chen and Cui, 2015). This means that individuals often use their own psychological state as a reference to infer the mental state of similar others or in-group members (Ames, 2004). Moreover, from the perspective of age and generations, individuals who are of the same generation have a similar social environment, life experiences, and shared values. These similarities become key to increasing in-group identification and favoritism (e.g., Henderson-King et al., 1997; Shi and Tang, 2015). Thus, when younger adults take their contemporaries’ perspective, they show the tendency of in-group favoritism based on their shared identity with in-group members. On the other hand, differences between self and the out-group become the main reason for intergroup conflicts and prejudice.

Finding the causes of intergroup differences and their mechanism may be an effective way to eliminate intergenerational conflicts. Thus, it is necessary to further investigate the mechanism behind this social phenomenon and to find interventions to improve intergenerational relationships. Study 2 aimed at solving this problem. It was found that negative stereotypes of older adults not only negatively predicted PT but also mediated the relationship between prior contact and PT, adding effective empirical evidence to the intergenerational relationship research field. Our results were consistent with those of Galinsky et al. (2008), which indicated that individuals utilize stereotypical information when taking out-group members’ perspectives. We also agreed with the idea of the “anchor” (Baryshevtsev et al., 2020); in that, when younger adults put themselves in the place of older adults, their original perceptual framework toward older adults is activated to meet the standard of PT. It should be noted that this activated perception framework is beneficial for the whole group of the older generation rather than for an individual. On this basis, the receiver of younger adults’ PT may not be the objective older adult target, but it instead is a reflection of a perceived figure that combines all the characteristics of the older generation that younger adults acknowledge. Then, younger adults imitate this represented figure’s mental state and perceive the negative traits from their stereotypes, which consequently makes it difficult for them to take the older individual’s perspective. In other words, younger adults’ failure to take older adults’ perspective probably results from the negative stereotypes they apply to the target of their PT. According to this assumption, negative stereotypes may block the ability of younger adults to perceive the target’s perspective objectively. As a consequence, younger adults may avoid contact with older adults because of the painful failure of their PT, which in turn may aggravate their negative stereotypes toward older people. This vicious circle is probably responsible for the phenomenon of the generation gap that was found in Study 1. The mechanism behind this relationship should be explored further in future research.

We also found that different levels of intergroup anxiety moderated the mediating relationships through similar paths. In the condition of low intergroup anxiety, only a low level of the antecedent (prior contact or stereotyping) had an impact on the dependent variable. In the condition of high intergroup anxiety, both high and low levels of the antecedent influenced the dependent variable in the same way. These results shed light on the influence of less prior contact toward stereotyping. Previous research has revealed that a lack of intergenerational contact results in more negative stereotypes toward older adults in younger adults’ perspective (e.g., Kwong See and Nicoladis, 2010; Jiang and Zhou, 2012; Lytle et al., 2020). The current research confirmed that point of view in finding that prior contact played a part in affecting stereotyping under the condition of low intergroup anxiety. Additionally, the result of a low level of stereotyping predicted a high level of PT under the condition of low intergroup anxiety; this was again consistent with the mediation relationship.

The results for the high intergroup anxiety condition were consistent with Stephan’s Intergroup Anxiety Theory (2014), which posits that the negative affections associated with intergroup anxiety could elicit thoughts about the difficulties of intergroup interactions. In this case, high intergroup anxiety increased younger adults’ negative stereotyping, whose antecedent was prior contact, and decreased their PT toward older adults, whose antecedent was stereotyping. It is notable that the quantity of contact seems to have little impact on younger adults’ stereotyping of older people when they feel more anxious during intergenerational encounters. In other words, in certain conditions, negative emotions, such as anxiety, may be a serious obstacle to the process of building positive intergenerational relationships even when there is ample previous contact. The same circumstances apply to stereotyping. Compared to those who have a higher level of negative stereotypes toward the older generation, younger adults who have a much lower level of stereotyping should have taken the older generation’s perspective more easily. Instead, the higher level of intergroup anxiety seems to suppress that positive effect of decreased stereotyping and manages to damage their ability to take the older generation’s perspective.

Another type of contact also effectively impacts younger adults’ PT toward age-based out-groups: imagined contact. This implies that imagined contact may influence PT through similar pathways to those of authentic contact. Study 3 asked younger adults to imagine positive encounters with older adults and thereby improved their PT. If we were to ask them to imagine a negative interaction with older adults, their PT may be disrupted. Future research might take a closer look at the influence of the valence of imagined contact on intergenerational PT.

A limitation of the present research is that a causal relationship could not be strictly inferred by the current correlation method. There also exists a lack of diversity of ethnic participant groups in the Chinese background, which leads to the limited generalizability of the findings of the present research. Future research might consider cultural differences in intergenerational relationships.

## Conclusion

In summary, the present research adds empirical evidence to our understanding of the impacts of prior contact, imagined contact, negative stereotypes of older adults, intergroup anxiety, and PT on intergenerational relationships. Chinese younger adults have more difficulties in taking the perspective of older adults than that of their peers. Younger adults who have had less contact with older adults had a higher level of negative stereotyping toward older adults in the condition of low intergroup anxiety, which led to decreased PT toward older adults. In addition, the positive-imagined intergenerational contact will effectively increase younger adults’ PT toward older adults.

## Data Availability Statement

The raw data supporting the conclusions of this article will be made available by the authors, without undue reservation.

## Ethics Statement

The studies involving human participants were reviewed and approved by Shaanxi Normal University Committee. The patients/participants provided their written informed consent to participate in this study.

## Author Contributions

YL conducted the studies and wrote the manuscript. XJ provided the valuable ideas and the collected data for the manuscript. YW provided the academical support and guidance in preparing the manuscript and granted the research. XZ helped in the collection of the research data. XY granted the research and provided the valuable advice with the manuscript. All authors contributed to the article and approved the submitted version.

## Conflict of Interest

The authors declare that the research was conducted in the absence of any commercial or financial relationships that could be construed as a potential conflict of interest.

## Publisher’s Note

All claims expressed in this article are solely those of the authors and do not necessarily represent those of their affiliated organizations, or those of the publisher, the editors and the reviewers. Any product that may be evaluated in this article, or claim that may be made by its manufacturer, is not guaranteed or endorsed by the publisher.
